# Nanotechnology-Enhanced Materials and Smart Implants in General Surgery: A Systematic Review of Human Clinical Outcomes

**DOI:** 10.7759/cureus.94703

**Published:** 2025-10-16

**Authors:** Abdulkreem Al-Juhani, Rodan Desoky, Norah Almutiri, Fayza Akil, Ghadeer M Almutawah, Faisal Alzahrani, Yahya M Alanazi, Hadeel A Al Mohammed, Mahmoud S Desoky

**Affiliations:** 1 Forensic Medicine, Forensic Medicine Center, Jeddah, SAU; 2 Surgery, Faculty of Medicine, King Abdulaziz University, Jeddah, SAU; 3 Medicine, College of Medicine, Alfaisal University, Riyadh, SAU; 4 Medicine, College of Medicine, Qassim University, Buraydah, SAU; 5 General Medicine, Batterjee Medical College, Jeddah, SAU; 6 Surgery, College of Medicine, King Faisal University, Al-Ahsa, SAU; 7 Otolaryngology - Head and Neck Surgery, King Saud University, Riyadh, SAU; 8 Medicine, Almaarefa University, Diriyah, SAU; 9 Internal Medicine and Gastroenterology, Sultan Bin Abdulaziz Humanitarian City, Riyadh, SAU

**Keywords:** general surgery, medical nanotechnology, nano-biotechnology, nanotechnology-enhanced materials, smart implants

## Abstract

Nanotechnology-enhanced materials and smart implants are emerging technologies that have shown promise in addressing various challenges in general surgery, including infection management, oncologic staging, and complication monitoring. In this systematic review, we aimed to examine the safety and efficacy of these technologies compared with conventional methods in patients undergoing general surgical procedures. A comprehensive search was conducted in MEDLINE (via PubMed), Embase, Scopus, Web of Science, the Cochrane Central Register of Controlled Trials, ClinicalTrials.gov, WHO ICTRP, IEEE Xplore/Inspec, and Google Scholar according to the Preferred Reporting Items for Systematic reviews and Meta-Analyses (PRISMA) guidelines, using standardized keywords related to nanotechnology, surgery, and clinical outcomes. The included studies were categorized according to intervention class and procedural type. Risk of bias was assessed using RoB 2 for randomized trials and ROBINS-I for nonrandomized studies, while outcome certainty was evaluated using GRADE. Nine eligible studies were included, comprising a total of 2,250 participants (infection prevention: 974; mapping/staging: 1,244; sensing: 32 drain-fluid samples). Due to heterogeneity among the studies, a meta-analysis could not be performed, and the results were therefore synthesized narratively. Silver-based topical creams and dressings demonstrated early bacterial clearance and improved wound healing. Carbon nanoparticle (CNP) suspensions enhanced lymph node detection sensitivity and preserved parathyroid hormone in thyroid cancer and nearly doubled sensitivity to micrometastatic lymph nodes in gastric cancer. Silver-ion-impregnated tracers in breast cancer showed comparable sensitivity for sentinel lymph node detection. Silver-ion-impregnated meshes showed no significant difference compared with conventional meshes. Overall, adverse effects were minimal or absent. In conclusion, nanotechnology-enhanced materials and smart implants show substantial potential to improve infection prevention, oncologic staging, and perioperative monitoring in general surgery, with minimal adverse effects. Silver-based topical antimicrobials, CNP suspensions, superparamagnetic iron oxide tracers, and innovative biosensor technologies were either comparable or superior to conventional alternatives. However, a clear knowledge gap regarding long-term outcomes and the heterogeneity of comparators highlights the need for larger, rigorously designed studies to guide clinical adoption.

## Introduction and background

Despite significant medical advances in general surgery, several clinical challenges persist. Three of the most important issues in the field today are accurate lymphatic staging, surgical site infections (SSIs), and delayed recognition of early postoperative complications [1,2]. SSIs remain among the most common health care-associated infections and are linked to increased length of hospital stay, higher health care costs, and greater morbidity. The growing threat of antimicrobial resistance (AMR) adds urgency to this issue, as bacterial resistance accounted for approximately 1.27 million deaths in 2019, compromising the safety of preoperative prophylaxis [3,4].

At the same time, postoperative complications such as anastomotic leakage are often detected late because of nonspecific clinical signs and delayed imaging. Similarly, accurate lymphatic staging still relies on radiotracers with or without blue dye mapping; however, these standards involve radiation exposure, logistical complexity, and limited accessibility [1,4]. Collectively, these gaps highlight the need for practical innovations, smarter materials, and advanced devices to prevent infections, identify complications earlier, and assist in surgical guidance.

Current standardized care protocols for pre- and postoperative infection prevention depend on timely antibiotic administration, antiseptic measures, and temperature and glucose control. However, the increasing reliance on antibiotics is undermined by AMR, and conventional wound dressings demonstrate variable efficacy in SSI prevention and wound healing. A recent multicenter clinical trial found that ionic-silver hydrofiber dressings reduced superficial SSIs compared with film dressings [5]. Another randomized trial reported improved healing and faster microbial clearance with silver nanoparticles [6].

Nodal mapping and detection play a crucial role in staging and treatment planning, particularly in breast cancer, where the current benchmark involves radioisotopes with or without blue dye. However, regulatory requirements, radiation exposure, and limited access can delay or complicate care. Nonradioactive tracers have recently emerged as viable alternatives: a multicenter noninferiority trial demonstrated that superparamagnetic iron oxide (SPIO) tracers are a practical substitute that avoids radiation exposure [7,8]. Carbon nanoparticles (CNPs) have also enhanced lymph node harvest and micrometastatic node detection, and in thyroid surgery, they have been shown to improve parathyroid preservation and nodal retrieval [9,10].

Early detection of postoperative complications such as anastomotic leaks remains a challenge because clinicians must often rely on nonspecific signs before ordering targeted imaging. This delay frequently leads to reinterventions and increases the risk of sepsis, morbidity, and prolonged hospitalization [4,11]. Novel bedside biosensors have shown promise as a complementary diagnostic pathway, and a non-electronic macromolecular network drain sensor has demonstrated excellent diagnostic performance for leak detection, although it has not yet been standardized [12].

Nanotechnology-enhanced materials and smart implants have been developed to address these clinical challenges while offering improved safety profiles. Several human studies have demonstrated their potential to reduce SSIs (e.g., ionic-silver hydrofiber dressings), replace or supplement radioisotopes for nodal mapping (e.g., SPIO and CNPs), and enable real-time perioperative monitoring (e.g., biosensors). However, existing evidence remains heterogeneous across procedures, comparators, and follow-up durations. In this review, we aim to consolidate current clinical findings to evaluate the safety, effectiveness, and translational potential of these emerging technologies in surgical practice.

## Review

Methodology

**Figure 1:**
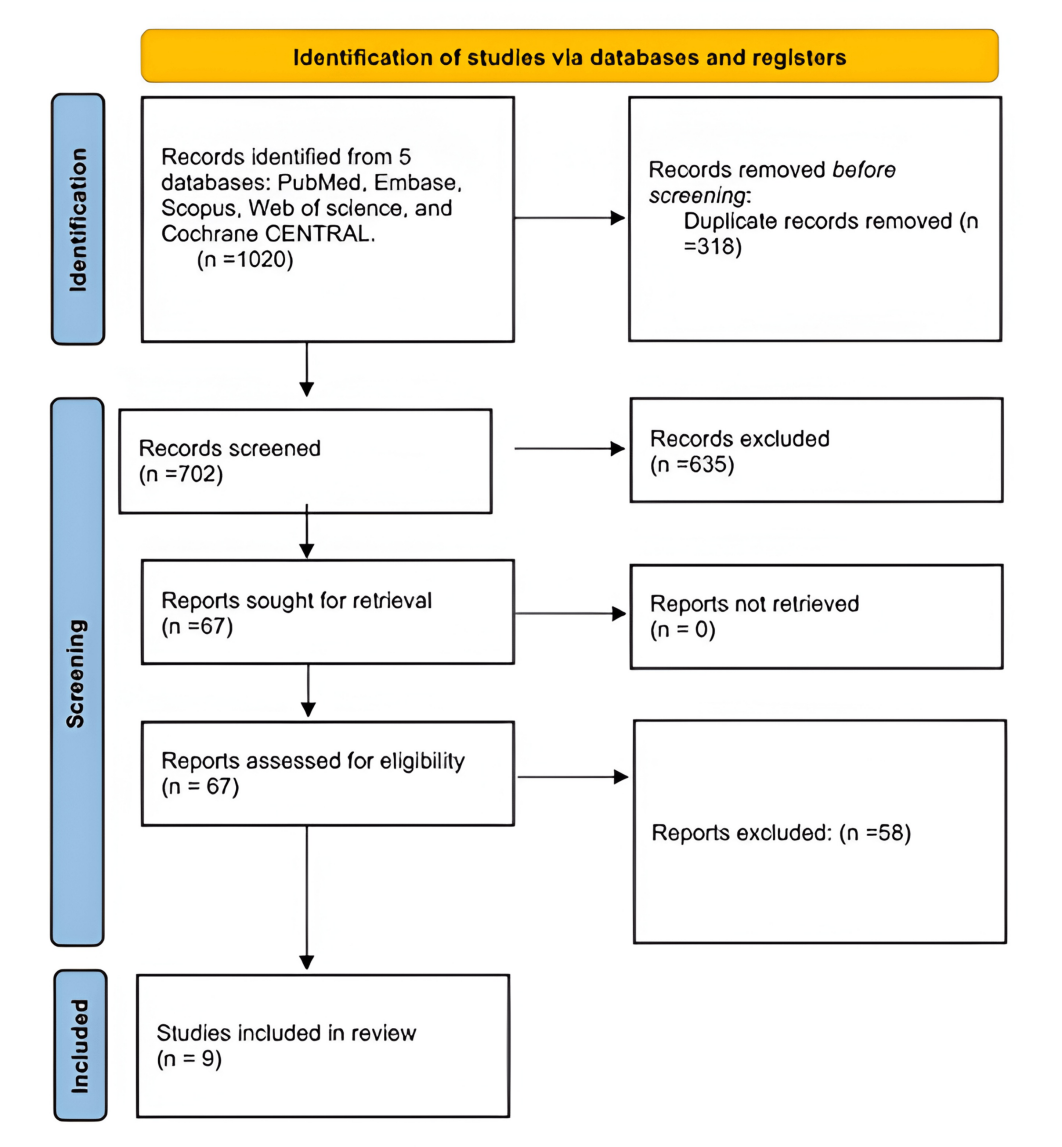
PRISMA flow diagram PRISMA, Preferred Reporting Items for Systematic reviews and Meta-Analyses

This review was designed according to a predefined methodology consistent with the Preferred Reporting Items for Systematic reviews and Meta-Analyses Protocols (PRISMA-P) and is reported in accordance with the PRISMA 2020 guidelines (Figure 1).

The eligibility criteria (PICO) are summarized in Table 1. The population included human patients of any age undergoing general surgical procedures, such as gastrointestinal surgery, hernia repair, endocrine or thyroid surgery, and soft-tissue treatments. The interventions comprised nanotechnology-enhanced materials or “smart” implants and devices utilized intraoperatively or perioperatively. These included nano-coated meshes or implants, nano-structured biomaterials, sensor-enabled drains or dressings, nanoparticle-mediated therapeutic drugs, and nano-tracers for surgical navigation. The comparators were conventional materials or devices and standard care that lacked nano-enhancement or intelligent features. The outcomes evaluated included at least one patient-relevant endpoint, such as surgical-site or implant infection, wound healing duration or complications, implant or device failure, identification or time-to-detection of complications, need for reoperation, and length of hospitalization.

**Table 1:** PICO search strategy

PICO element	Scope	Keywords/synonyms/MeSH terms
Population (P)	Patients undergoing general surgery (adults or children) - limited to general/abdominal surgery	Keywords: “general surgery” OR “abdominal surgery” OR “gastrointestinal surgery” OR “hernia repair” OR “soft tissue resection” OR “laparotomy” OR “laparoscopic surgery”. MeSH: “General Surgery”[Mesh] OR “Abdominal Surgery”[Mesh] OR “Digestive System Surgical Procedures”[Mesh]
Intervention (I)	Nanotechnology-enhanced surgical materials, devices, smart implants, nanoparticle therapies, or smart dressings	Keywords: nanotechnology OR nanomaterials OR nanoparticles OR “nano-enhanced” OR “nanostructured biomaterials” OR “antimicrobial coating” OR “nanoparticle-coated mesh” OR “smart implant” OR biosensor OR “smart device” OR “smart wound dressing” OR “nano-drug delivery”. MeSH: “Nanotechnology”[Mesh] OR “Nanostructures”[Mesh] OR “Nanoparticles”[Mesh] OR “Biomaterials”[Mesh] OR “Drug Delivery Systems”[Mesh]
Comparator (C)	Conventional surgical materials or standard surgical care	Keywords: “standard care” OR “conventional mesh” OR “standard surgical materials” OR “routine practice” OR “traditional implant”. MeSH: “Surgical Procedures, Operative”[Mesh] OR “Standard of Care”[Mesh]
Outcomes (O)	Clinical patient outcomes, including infection rates, wound healing, implant success or failure, complication detection, and morbidity or recovery	Keywords: “surgical site infection” OR “postoperative infection” OR “wound healing” OR “wound complication” OR “mesh failure” OR “implant failure” OR “hernia recurrence” OR “complication detection” OR “anastomotic leak” OR “patient outcome” OR morbidity OR recovery. MeSH: “Surgical Wound Infection”[Mesh] OR “Wound Healing”[Mesh] OR “Treatment Outcome”[Mesh] OR “Postoperative Complications”[Mesh]

Study designs included randomized controlled trials, controlled clinical trials, and comparative observational studies. Conference abstracts were admissible if they provided adequate outcome data. Exclusion criteria comprised non-human studies; specialties unrelated to general surgery that did not provide extractable general surgery data; single-arm reports without a relevant comparator; and studies that solely presented surrogate or device performance indicators. No restrictions regarding publication date or language were applied.

Sources of information and search methodology: The search was conducted through MEDLINE (via PubMed), Embase, Scopus, Web of Science, and the Cochrane Central Register of Controlled Trials, from database inception to the final search date. To capture transdisciplinary and grey literature, additional searches were performed in ClinicalTrials.gov, WHO ICTRP, IEEE Xplore/Inspec, Google Scholar, and major conference proceedings. Reference lists and forward citations of the included studies were also examined. Search strategies, developed in collaboration with a librarian, integrated controlled vocabulary and keywords across three domains: nanotechnology/smart materials, surgery, and clinical outcomes.

Selection of Studies and Management of Data

Duplicate studies were removed using a reference manager before importing records into a screening platform. Two reviewers independently screened titles and abstracts, followed by full-text assessments based on the eligibility criteria. Any discrepancies were resolved through consensus or adjudication by a third reviewer. The rationale for full-text exclusions was documented, and study progression was illustrated in a PRISMA flow diagram. A version-controlled repository maintained the methodology, screening forms, data extraction templates, and audit logs.

Data extraction and components: Two reviewers independently extracted data using a piloted, standardized form. Extracted data included study characteristics (authors, year, country, setting, funding, and conflicts of interest); study design (trial or observational, single-center, or multicenter), follow-up duration; population details (age, sex, indications, comorbidities, and procedure type); intervention details (nano feature, material or device type, coating composition, particle characteristics, sensor type, dose or exposure, timing, and implementation); comparator details; outcome definitions and measurement windows; and numerical results (events and denominators for dichotomous outcomes; means and standard deviations or medians and interquartile ranges for continuous outcomes; and HRs for time-to-event outcomes). Adverse events were also recorded. Authors were contacted for clarification or to obtain missing data when necessary.

Risk of Bias

Two reviewers independently assessed the risk of bias using RoB 2 for randomized trials and ROBINS-I for nonrandomized studies (or the Newcastle-Ottawa Scale when applicable). Domain-level and overall assessments informed interpretation and sensitivity analyses. The certainty of evidence for key outcomes was evaluated using GRADE, considering study limitations, inconsistency, indirectness, imprecision, and potential publication bias.

No quantitative aggregation was conducted. Instead, a systematic narrative synthesis was performed, categorizing studies by intervention class (e.g., antimicrobial nano-coatings, nano-tracers, sensor-enabled devices, and nanoparticle administration) and by procedural type. For each outcome, the direction and magnitude of effects were described using the estimates from each study (risk or ORs, mean differences, and HRs with 95% CIs when available), explicitly avoiding vote counting based on statistical significance. In cases of heterogeneous outcome measures, ranges and medians were reported, correlating findings with study design, risk of bias, and clinical context. Planned stratified summaries (e.g., adult versus pediatric populations; clean versus contaminated operations; open versus minimally invasive procedures; and study design or risk-of-bias strata) and sensitivity considerations (e.g., abstracts-only reports) were addressed narratively. Funnel plots and tests for small-study effects were not conducted.

Ethics and Transparency

This review synthesizes previously published data; therefore, ethics approval was not required. Comprehensive search methods, a PRISMA flow diagram, risk-of-bias assessments, and, where permissible, de-identified data extraction files and analytical materials were included to enhance reproducibility.

Results

Overview of the Evidence

Nine studies met the inclusion criteria, comprising four randomized controlled trials, two prospective multicenter comparative studies, one retrospective cohort study, one case series with historical controls, and one diagnostic validation study. These studies addressed three main indications: infection prevention (silver-based materials), lymphatic mapping and oncologic staging (CNPs and SPIO), and early complication detection (macromolecular network biosensor). In total, 2,250 participants or samples were included across studies (infection prevention: 974; mapping/staging: 1,244; sensing: 32 drain-fluid samples). Variability in interventions, comparators, and outcome assessment periods precluded meta-analysis; therefore, results were synthesized narratively and organized by indication (Table 2).

**Table 2:** Design and methods snapshot (by intervention class) AL, anastomotic leak; CNS(I), carbon nanoparticle suspension (injection); ICG, indocyanine green; mITT, modified intention to treat; PTH, parathyroid hormone; RCT, randomized controlled trial; RLN, recurrent laryngeal nerve; SLNB/SLN, sentinel lymph node (biopsy); SSI, surgical site infection; TT, total thyroidectomy; UL, unilateral lobectomy

Study (year)	Setting/population	Design	N (analyzed)	Intervention (class)	Comparator	Primary outcome(s)	Follow up
Pathi et al. (2024) [10]	Infected wounds (mixed)	RCT, open-label, parallel	86 (43 vs. 43)	Silver nanoparticle topical cream (antimicrobial nanotech)	Mupirocin	Bacterial clearance (day 5); complete wound healing (day 28)	28 days
Kosugi et al. (2024) [11]	Elective GI surgery incisions	RCT	865 (427 vs. 438)	Aquacel Ag Hydrofiber (ionic silver dressing)	Film dressing	Superficial SSI ≤30 days (UMIN 000043081)	30 days
Zhao et al. (2023) [12]	Papillary thyroid cancer; thyroidectomy (UL and TT subgroups)	RCT, multicenter, blank-controlled	569 randomized	CNS tracer	Control (no tracer)	PTH; total LNs; tiny LNs; parathyroid recognition/retention; RLN	Intra-op; POD1; 1 mo
Tian et al. (2025) [13]	Resectable gastric cancer (cT1-4a N0/+ M0)	RCT, phase 3, open-label	96 randomized; 90 mITT (46 vs. 44)	CNSI tracer (pre-op endoscopic injection)	ICG fluorescence tracer	Retrieved LNs (primary); micro LNs; diagnostic value; 3 y DFS/OS planned	30-day morbidity; long-term planned
Olona et al. (2025) [14]	Incisional hernia with clean-contaminated wounds (ostomy reversal/bowel resection)	Case series, retrospective vs. matched historical controls	12 (silver mesh) vs. 11 (polypropylene)	Silver ion-impregnated polypropylene mesh	Conventional polypropylene mesh	SSI; morbidity; mesh explant	Discharge; 30 days; 12 mo
Jessernig et al. (2024) [15]	GI surgery patients - drain fluid samples	Diagnostic evaluation of clinical samples	32 samples	Naked-eye macromolecular network sensors (smart biosensor)	Reference AL status	Diagnostic accuracy (ROC AUC) for early AL	Perioperative samples
Vidya et al. (2023) [16]	Early breast cancer - SLNB	Prospective multicenter comparative	107	SPIO magnetic tracer (Sienna+)	Tc 99m ± blue dye	SLN identification rate; non-inferiority	Perioperative
Alvarado et al. (2019) [17]	Early breast cancer - SLNB	Prospective multicenter non-inferiority	146	SPIO magnetic tracer (Magtrace™)	Tc 99m + blue dye	SLN identification; non-inferiority margin	Perioperative
Zhang et al. (2018) [18]	Early breast cancer - SLNB	Retrospective single-center cohort	332	CNS tracer	ALND verification subset	SLN identification; diagnostic accuracy metrics	Perioperative

Infection Prevention

Two randomized studies and one case series evaluated silver-based interventions for infection prevention. In an RCT involving gastrointestinal incisions (n = 865), an ionic-silver hydrofiber dressing significantly reduced superficial SSIs within 30 days compared with a standard film dressing (6.8% vs. 11.4%; adjusted OR, 0.60; 95% CI, 0.37-0.99), representing an absolute risk reduction of 4.6% (46 per 1,000). In another trial of infected wounds (n = 86), a topical silver nanoparticle cream demonstrated superior bacterial clearance by day 5 (86% vs. 65.1%) and complete wound healing by day 28 (81.4% vs. 37.2%) compared with the control. A smaller clean-contaminated incisional hernia series (12 vs. 11) reported no SSIs (0/12) with silver-impregnated mesh, in contrast to 3/11 cases (27.3%) in the historical control group (p = 0.052); no mesh explants were required. None of the studies reported device- or drug-related adverse effects. The summarized findings are presented in Table 3.

**Table 3:** Key effect metrics and safety AE, adverse event; AL, anastomotic leak; AUC, area under the ROC curve; Ca²⁺, serum calcium concentration; CNS, carbon nanoparticle suspension; CNSI, carbon nanoparticle suspension injection; COI, conflict of interest; DFS, disease free survival; FNR, false negative rate; ICG, indocyanine green (fluorescent tracer); LN, lymph node; micro LNs, micro lymph nodes (small lymph nodes retrieved on detailed sorting); mITT, modified intention to treat; N/A, not applicable; NIR, near infrared; NR, not reported (in the abstract/text); OS, overall survival; PTH, parathyroid hormone; RCT, randomized controlled trial; RI, radioisotope (e.g., technetium labeled tracer); RLN, recurrent laryngeal nerve; ROC, receiver operating characteristic; SLN, sentinel lymph node; SLNB, sentinel lymph node biopsy; SPIO, superparamagnetic iron oxide (magnetic tracer); SSI, surgical site infection; TT, total thyroidectomy; UL, unilateral lobectomy (thyroid); Δ, absolute difference (“delta”) between groups

Study (design; n)	Population/procedure	Primary effect (quantitative)	Secondary findings (concise)	Safety/AEs	Key limitations
Pathi et al. (2024) [10] - RCT, open-label; n = 86	Infected wounds (mixed etiologies); topical silver nanoparticle cream vs. mupirocin	Bacterial clearance at day 5: 86% vs. 65.1% (p = 0.023). Complete healing by day 28: 81.4% vs. 37.2% (p ≤ 0.001)	Faster wound improvement across visits (Bates-Jensen tool) reported	No local/systemic AEs observed in either arm	Single-center; open-label; co-interventions (systemic antibiotics) allowed; short (28-day) follow-up
Kosugi et al. (2024) [11] - RCT; n = 865	Elective GI surgery; Aquacel Ag Hydrofiber vs. film dressing	Superficial SSI ≤30 d: 6.8% vs. 11.4% (p = 0.019); adjusted OR 0.60 (95% CI 0.37-0.99; p = 0.044)	Benefit concentrated in the lower GI subgroup (p = 0.042); no difference in the upper GI subgroup	AEs not detailed in abstract; routine peri-op antibiotics standardized (protocol in full text). No safety signal noted	Single-hospital RCT; dressing vs. film only; outcome limited to superficial SSI; blinding not feasible
Zhao et al. (2023) [12] - RCT, multicenter; n = 569	Papillary thyroid cancer; thyroidectomy; CNPs (CNS) vs. control	UL subgroup: ↑ total LNs (p < 0.0001) and ↑ tiny LNs (p = 0.0268). TT subgroup: higher 1-month PTH with CNS (p = 0.0368)	No significant differences in serum Ca²⁺; parathyroid preservation and RLN injury rates similar; RLN protection assessed as secondary	No drug-related AEs or injection complications recorded	Endpoint heterogeneity across UL vs. TT; surgeon blinding impossible; clinical significance of “tiny LNs” yield requires context
Tian et al. (2025) [13] - RCT; modified ITT n = 90	Radical gastrectomy; CNSI vs. ICG tracer	Retrieved LNs (mean): 69.8 vs. 53.6 (p < 0.001). Micro LNs: 19.9 vs. 11.6 (p = 0.001)	Diagnostic metrics for metastasis: higher sensitivity for micro LNs with CNSI; workflow note - LN sorting faster with CNSI vs. NIR ICG	30-day complications: 10.9% vs. 11.4% (p = 0.94); detailed breakdown shows no mortality; no tracer injection complications	Open-label; modest sample; short-term outcomes (DFS/OS planned but not yet available); device/platform differences (robotic NIR) may confound
Olona et al. (2025) [14] - case series vs. historical control; n = 12 (silver mesh) vs. 11 (prior PP mesh)	Clean-contaminated incisional hernia repair; silver-impregnated polypropylene mesh	SSI: 0/12 vs. 3/11 (27.3%) in prior series; p = 0.052 (borderline). Explants: 0	No significant differences in other follow-up variables; mixed repair techniques (mostly retromuscular)	No SSI and no explants in silver mesh cohort over ~12-month mean follow-up; specific AEs otherwise NR in abstract	Small, single-center, nonrandomized; historical comparator; short follow-up; potential selection/surgeon bias
Jessernig et al. (2024) [15] - diagnostic study; n = 32 patients’ drain samples	Post-op GI surgery; visible, non-electronic macromolecular network sensors for enzyme leak detection	ROC AUC for AL detection: 1.0 on clinical samples (proof of concept)	Continuous bedside monitoring concept; naked-eye color shift for amylase/protease signature	Not applicable (no patient implantation/intervention in this study); COI: inventorship on patent declared	Early-phase diagnostic work; small sample; clinical impact (time to detection, outcomes) not yet tested in trials
Vidya et al. (2023) [16] - prospective, multicenter; n = 107 analyzed	Early breast cancer; SLNB; SPIO vs. radioisotope ± blue dye	Per-patient SLN detection: 98.13% (SPIO) vs. 92.26% (standard). Per-node: 93.07% vs. 96.53%. All 31 node-positive cases detected by both	Demonstrated feasibility; concordance shown	AEs NR in abstract (focus on detection performance)	Not randomized head-to-head (both tracers used sequentially in the same patients); detection emphasis; limited safety reporting in the abstract
Alvarado et al. (2019) [17] - multicenter non-inferiority; n = 146	Early breast cancer; SLNB; Magtrace (SPIO) vs. technetium-99m + blue dye	Per-patient detection: 99.3% vs. 98.6%. Per-node: 94.3% vs. 93.5%. Δ 0.8%, 95% CI lower bound −2.1% → met non-inferiority	All malignant nodes found by standard were identified by SPIO; high concordance	AEs NR in abstract (performance trial)	Open-label procedural trial; safety outcomes not detailed in abstract; no long-term outcomes
Zhang et al. (2018) [18] - retrospective cohort; n = 332	Early breast cancer; SLNB; CNS	SLN identification: 99.1% (329/332). Sensitivity: 95.9%. Specificity: 100%. FNR: 4.1%	Mean SLNs retrieved: 2.6; high diagnostic accuracy comparable to standards	Anaphylaxis, necrosis, local inflammation: 0; skin staining: 41.9% at median 13.2-month follow-up	Single-center retrospective; no RI/blue dye control arm; cosmetic staining common

Lymphatic Mapping and Oncologic Staging

The evidence base comprised two randomized controlled trials (thyroidectomy and radical gastrectomy), two prospective multicenter studies on breast cancer sentinel lymph node biopsy (SLNB) comparing SPIO with radioisotope mapping (with or without blue dye), and one retrospective cohort study evaluating CNS in breast cancer.

In the FUTURE 01 trial (mITT n = 90) for radical gastrectomy, injection of CNP suspension (CNS) increased total lymph node yield (mean 69.8 vs. 53.6) and micro lymph node count (19.9 vs. 11.6), while 30-day postoperative complication rates were comparable between groups (10.9% vs. 11.4%). In thyroidectomy (n = 569), CNS improved nodal retrieval in unilateral lobectomy subanalyses and was associated with higher one-month postoperative PTH levels after total thyroidectomy; serum calcium concentrations and RLN injury rates were unchanged in the reported abstract.

For breast cancer SLNB, two prospective multicenter RCTs, such as SMART (n = 107) and SentimagIC (n = 146), evaluated SPIO tracers. Both studies demonstrated non-inferiority to conventional radioisotope-based mapping, achieving per-patient SLN detection rates of 98.1% versus 92.3% (SMART) and 99.3% versus 98.6% (SentimagIC). All malignant nodes identified through standard techniques were also detected by SPIO.

A retrospective cohort of breast cancer patients (n = 332) using CNS for SLNB reported SLN identification in 99.1% of cases, sensitivity of 95.9%, specificity of 100%, and a false negative rate of 4.1%. Skin staining occurred in 41.9% of patients at a median of approximately 13 months post-procedure. No significant tracer-related adverse effects were reported in the mapping studies.

Early Complication Sensing

A diagnostic validation study (n = 32 clinical drain samples) employing a visual, non-electronic macromolecular network sensor achieved an AUC of 1.00 for detecting biochemical signatures of anastomotic leaks. Patient-level clinical outcomes such as time-to-detection, reoperation, and infection rates were not evaluated. No device-related adverse events were reported in this preliminary investigation (Table 3).

Safety

Across studies, no significant adverse effects were associated with silver dressings or creams, CNS/CNSI injections, or SPIO tracers. The only recurring safety issue was cosmetic skin discoloration related to CNS use in breast SLNB, observed in approximately 42% of cases. Postoperative morbidity within 30 days did not differ significantly between CNSI and ICG in gastrectomy procedures (Table 3).

Risk of Bias

All four randomized trials were judged as having “some concerns” overall according to the RoB 2 tool. The non-randomized silver-mesh series was rated “serious” overall risk using ROBINS-I. Diagnostic accuracy evaluations using QUADAS-2 classified the SPIO studies (SMART, SentimagIC) as “low risk,” whereas the CNS breast cohort and the macromolecular sensor study were both rated “high risk.” These risk-of-bias assessments warrant cautious interpretation, particularly for non-randomized and early-phase diagnostic studies. The detailed assessments are presented in Table 4, Table 5, and Table 6.

**Table 4:** Randomized trials - RoB 2 (domain-level judgments only) Domains: Randomization process (R); Deviations from intended interventions (D); Missing outcome data (M); Measurement of the outcome (O); Selection of the reported result (S); Overall assessment

Study	R	D	M	O	S	Overall
Pathi et al. (2024) [10]	Some concerns	Some concerns	Low	Some concerns	Some concerns	Some concerns
Kosugi et al. (2024) [11]	Low	Some concerns	Low	Low	Some concerns	Some concerns
Zhao et al. (2023) [12]	Low	Some concerns	Low	Some concerns	Some concerns	Some concerns
Tian et al. (2025) [13]	Low	Some concerns	Some concerns	Some concerns	Some concerns	Some concerns

**Table 5:** Non-randomized interventional study - ROBINS-I (domain-level judgments only) Domains: Confounding (C); Selection of participants (S); Classification of interventions (Cl); Deviations from intended interventions (D); Missing data (M); Measurement of outcomes (O); Selection of the reported result (R); Overall assessment

Study	C	S	Cl	D	M	O	R	Overall
Olona et al. (2025) [14] (silver-impregnated mesh)	Serious	Serious	Low	Moderate	Moderate	Moderate	Moderate	Serious

**Table 6:** Diagnostic accuracy studies - QUADAS (risk of bias and applicability judgments only) Risk of bias domains: Patient selection (PS); Index test (IT); Reference standard (RS); Flow and timing (FT); Overall risk of bias (RoB) Applicability concerns: PS A, IT A, RS A

Study	Risk of Bias - PS	IT	RS	FT	Overall RoB	Applicability - PS A	IT A	RS A
Jessernig et al. (2024) [15] (Advanced Science; macromolecular network sensor)	High	Unclear	Unclear	Unclear	High	High	High	Unclear
Vidya et al. (2023) [16] (The Surgeon; SMART, SPIO vs. RI)	Low	Low	Low	Low	Low	Low	Low	Low
Alvarado et al. (2019) [17] (Annals of Surgical Oncology; SentimagIC, SPIO vs. RI + dye)	Low	Low	Low	Low	Low	Low	Low	Low
Zhang et al. (2018) [18] (World Journal of Surgical Oncology; CNS for SLN mapping)	High	Low	Low	Unclear	High	Low	Low	Low

Synthesis Statement

The collective evidence supports the use of silver-based approaches for reducing superficial SSIs and accelerating wound healing in selected settings. It also confirms that SPIO is a non-inferior alternative to radioisotopes for sentinel lymph node mapping and highlights the benefits of CNPs in improving nodal yield and preserving parathyroid function, without introducing new safety concerns. Biosensor technology shows excellent diagnostic accuracy; however, further clinical studies are required to establish its impact on patient-relevant outcomes.

Discussion

Our systematic review aimed to evaluate the safety, effectiveness, and therapeutic potential of nanotechnology-enhanced materials and smart implants in general surgery based on human clinical outcomes. The nine included clinical studies demonstrated the significance of nanotechnology across diverse surgical applications. The evidence showed that silver-based materials markedly reduced superficial SSIs and promoted wound healing [5,6,13-16]. SPIO demonstrated comparable effectiveness to conventional mapping techniques [17-20]. CNSs in SLNB showed high identification rates with no significant adverse effects [9,15,16]. One preliminary study using a non-electronic macromolecular network sensor showed the potential to identify anastomotic leakage. Collectively, these findings highlight the promising benefits of these technologies for improving human clinical outcomes in the future [21].

Regarding the effectiveness of silver-based technology, the literature has shown encouraging results. Metal-based nanoparticles possess strong antibacterial properties, providing high potential for treating bacterial infections [22]. A recent randomized clinical trial comparing mupirocin with Kadermin, a silver nanoparticle-based cream, reported significant differences in wound healing and bacterial clearance of culture-positive infected wounds [23]. Similarly, when compared with standard film dressings, Aquacel Ag Hydrofiber dressings reduced the incidence of superficial SSIs by approximately 40% [24]. Metal-based nanoparticles thus show considerable promise as antibacterial agents for infection management [25].

Previous single-center and sample-based studies have demonstrated that CNPs can enhance lymph node detection in colorectal cancer [25,26] and papillary thyroid cancer [27,28]. In contrast, another study found no significant benefit in lymph node dissection when using CNPs [29]. However, our findings suggest that CNS may aid in detecting a higher number of lymph nodes, particularly micrometastatic or small lymph nodes, in papillary thyroid cancer, potentially enabling more radical dissections. Aside from minor skin staining noted in one study, CNS demonstrated comparable efficacy to traditional methods for lymph node retrieval and improved parathyroid preservation.

Two studies reported that SPIO-guided SLNB is a viable alternative for early breast cancer, achieving identification rates comparable to the standard dual technique using radioisotopes and blue dye. This suggests SPIO could safely replace the gold standard for sentinel lymph node mapping [30]. The use of SPIO during mastectomy for uncomplicated conditions also spared most patients unnecessary axillary surgery and its associated morbidities [31]. Multiple studies utilizing various tools for early detection of anastomotic leaks underscore the importance of timely diagnosis and support the potential value of innovative biosensor approaches [32].

Despite the small sample sizes, short follow-up durations, and heterogeneous outcomes among included studies, our systematic review represents a preliminary but important step in assessing the clinical outcomes of nanotechnology-enhanced materials and smart implants in general surgical practice. Strengths of this review include adherence to PRISMA 2020 guidelines, the use of multiple reviewers, standardized data collection, and a structured evaluation of bias. Additionally, our exclusive focus on human clinical studies enhances the practical relevance of the findings.

One of the greatest challenges in modern medicine is the rapid emergence of AMR among common pathogens [32]. In this context, silver-based topical therapies and dressings offer a promising alternative, providing broad-spectrum antibacterial activity and the potential to reduce infection rates and healthcare utilization. Both CNS and SPIO also serve as effective alternatives to traditional radioisotopes and dyes. CNS demonstrated clear benefits, including improved micrometastatic lymph node retrieval, while SPIO yielded outcomes comparable to conventional radioisotopes, with the added advantage of eliminating radiation exposure.

Although still in preliminary stages, biosensor technologies represent a major shift toward real-time, early detection of postoperative complications, including anastomotic leakage. Such advancements may enable earlier intervention and potentially reduce morbidity. However, further large-scale, multicenter randomized controlled trials with extended follow-up periods are required to evaluate delayed complications. Specifically, for biosensors, future prospective trials should emphasize patient-centered outcomes such as leak detection timing, reoperation rates, and postoperative morbidity.

## Conclusions

The findings underscore that nanotechnology-enhanced materials and intelligent implants hold substantial potential to transform general surgical practice. These innovations demonstrate promise in three key areas: reducing the risk of SSIs, improving the precision of lymphatic mapping in thyroid, gastric, and breast procedures, and enabling early detection of postoperative complications. The reviewed studies indicate that these approaches can produce outcomes comparable to or exceeding current standards, with few adverse effects reported. However, existing trials remain limited by small sample sizes, short follow-up durations, and heterogeneity in comparators and outcome measures. Therefore, large-scale, multicenter studies with extended follow-up are essential to confirm their safety, assess long-term clinical benefits, and establish standardized protocols for widespread clinical implementation.
